# Impact of Caffeine Consumption on Type 2 Diabetes-Induced Spatial Memory Impairment and Neurochemical Alterations in the Hippocampus

**DOI:** 10.3389/fnins.2018.01015

**Published:** 2019-01-09

**Authors:** João M. N. Duarte, Cecilia Skoug, Henrique B. Silva, Rui A. Carvalho, Rolf Gruetter, Rodrigo A. Cunha

**Affiliations:** ^1^CNC-Center for Neuroscience and Cell Biology, University of Coimbra, Coimbra, Portugal; ^2^Laboratory for Functional and Metabolic Imaging, École Polytechnique Fédérale de Lausanne, Lausanne, Switzerland; ^3^Department of Experimental Medical Science, Faculty of Medicine, Lund University, Lund, Sweden; ^4^Wallenberg Centre for Molecular Medicine, Lund University, Lund, Sweden; ^5^Department of Life Sciences, Faculty of Science and Technology, University of Coimbra, Coimbra, Portugal; ^6^Department of Radiology, University of Lausanne, Lausanne, Switzerland; ^7^Department of Radiology, University of Geneva, Geneva, Switzerland; ^8^Faculty of Medicine, University of Coimbra, Coimbra, Portugal

**Keywords:** insulin, adenosine, caffeine, neuroprotection, synaptic dysfunction, gliosis, glucose, metabolic profiling

## Abstract

Diabetes affects the morphology and plasticity of the hippocampus, and leads to learning and memory deficits. Caffeine has been proposed to prevent memory impairment upon multiple chronic disorders with neurological involvement. We tested whether long-term caffeine consumption prevents type 2 diabetes (T2D)-induced spatial memory impairment and hippocampal alterations, including synaptic degeneration, astrogliosis, and metabolic modifications. Control Wistar rats and Goto-Kakizaki (GK) rats that develop T2D were treated with caffeine (1 g/L in drinking water) for 4 months. Spatial memory was evaluated in a Y-maze. Hippocampal metabolic profile and glucose homeostasis were investigated by ^1^H magnetic resonance spectroscopy. The density of neuronal, synaptic, and glial-specific markers was evaluated by Western blot analysis. GK rats displayed reduced Y-maze spontaneous alternation and a lower amplitude of hippocampal long-term potentiation when compared to controls, suggesting impaired hippocampal-dependent spatial memory. Diabetes did not impact the relation of hippocampal to plasma glucose concentrations, but altered the neurochemical profile of the hippocampus, such as increased in levels of the osmolites taurine (*P* < 0.001) and *myo*-inositol (*P* < 0.05). The diabetic hippocampus showed decreased density of the presynaptic proteins synaptophysin (*P* < 0.05) and SNAP25 (*P* < 0.05), suggesting synaptic degeneration, and increased GFAP (*P* < 0.001) and vimentin (*P* < 0.05) immunoreactivities that are indicative of astrogliosis. The effects of caffeine intake on hippocampal metabolism added to those of T2D, namely reducing *myo*-inositol levels (*P* < 0.001) and further increasing taurine levels (*P* < 0.05). Caffeine prevented T2D-induced alterations of GFAP, vimentin and SNAP25, and improved memory deficits. We conclude that caffeine consumption has beneficial effects counteracting alterations in the hippocampus of GK rats, leading to the improvement of T2D-associated memory impairment.

## Introduction

Metabolic syndrome and diabetes *mellitus* affect brain function and increase the risk of age-related cognitive impairment, vascular dementia, and Alzheimer's disease (Frisardi et al., 2010; Duarte, 2015; Moheet et al., 2015). Diabetes conditions are particularly associated with atrophy of the hippocampus (Convit et al., 2003; Gold et al., 2007). We and others have reported that experimental diabetic conditions cause synaptic degeneration (Duarte et al., 2006, 2009a, 2012), increase astrocytic reactivity and proliferation (Saravia et al., 2002; Baydas et al., 2003; Duarte et al., 2012), and change metabolism (Duarte et al., 2009a; Girault et al., 2018) in the hippocampus. As important as understanding the mechanisms of diabetes-induced hippocampal alterations leading to memory impairment, is the design of novel strategies to prevent such degeneration. The neuromodulation system operated by adenosine is altered in diabetes, with reduced density of adenosine A_1_ receptors (A_1_Rs) and increased density of adenosine A_2A_ receptors (A_2A_Rs) in membranes from the hippocampus (Duarte et al., 2006, 2012). Caffeine is a widely consumed non-selective antagonist of adenosine receptors (Fredholm et al., 1999), and both caffeine and selective adenosine A_2A_R antagonists affect performance in learning and memory tasks (Takahashi et al., 2008; Cunha, 2016) and afford neuroprotection upon chronic brain insults (Cunha, 2005). In addition, caffeine may reduce the risk of developing glucose-intolerance and diabetes severity (e.g., van Dam and Hu, 2005; Greenberg et al., 2006; Higdon and Frei, 2006). We previously reported that caffeine consumption ameliorates diabetes-induced hippocampal degeneration and prevents diabetes-associated memory deficits in insulin-deficient rats (Duarte et al., 2009a) and in a mouse model of obesity-associated type 2 diabetes (T2D) (Duarte et al., 2012). The different etiology of lean T2D prompted us to investigate the effect of long-term caffeine intake on alterations in the hippocampus of Goto-Kakizaki (GK) rats, an animal model of non-obese T2D that was produced by selective breeding of non-diabetic Wistar rats which displayed high plasma glucose levels in oral glucose tolerance tests (Girault et al., 2018, and references therein). In this study, we tested the hypothesis that caffeine exposure ameliorates T2D-induced alterations of hippocampal metabolism, degeneration of synapses and astrogliosis, as well as concomitant spatial memory impairment.

## Methods

### Animals

Animals were handled according to Swiss and Portuguese guidelines for the use of experimental animals, and authorized by the respective local ethics committees (EXPANIM-SCAV and ORBEA). Male GK rats, which spontaneously develop insulin resistance, and control Wistar-Hannover-Galas rats were obtained from Taconic (Lille Skensved, Denmark), or from the colony kept at the animal house of the Faculty of Medicine of the University of Coimbra (for electrophysiology recordings). We used a total of 22 GK rats and 22 Wistar rats. All the animals were maintained with food and water *ad libitum*. When tested, caffeine was administered in the drinking water at 1 g/L from 2 to 6 months of age (4 month period). Thus, the present experimental design included four animal groups: control Wistar, caffeine-treated Wistar, diabetic GK, and caffeine-treated GK. Body weight and caffeine consumption were monitored throughout the treatment period. Glycaemia was measured monthly in a 2 μL blood sample collected by tail pricking, using a glucose oxidase-based glucometer (Ascencia Contour, Bayer, Switzerland). At 2 and 4 months of caffeine exposure, blood samples (100 μL) were taken from the tail vein under brief isoflurane anesthesia (2% in oxygen) for determination of serum caffeine and/or insulin concentrations.

### Behavioral Tasks

Exploratory behavior and locomotor activity were evaluated in a square open-field arena of 34 × 34 cm with 30 cm high, which was divided in 4 squares of 17 × 17 cm. The animals were placed in the central area of the arena and allowed to explore it over 5 min in the dark. The number of crossings of the squares and the number of rearing movements with forepaws were recorded. Rearing with the forepaws pressed against the walls was not considered.

Spontaneous alternation was observed in a Y-maze constructed in black Plexiglas, with three arms measuring 35 cm long, 9 cm wide and 30 cm height, and converging to equal angles, which was placed in a dim-illuminated room (12 lux) with large visual cues hanged on the walls. The animals were placed at the bottom of one arm of the Y-maze and allowed to explore freely all three arms for a single 8 min session in the dark. The measured spontaneous alternation behavior was used to assess hippocampal-dependent spatial memory (Lalonde, 2002). If the rat remembers the arm it has just explored, it will therefore enter one of the other arms of the maze. Complete spontaneous alternations were defined as successive entries into the three arms, and were expressed as fraction of the possible alternations in the respective test. In addition to the open field test, the number of entries in the arms of the maze also allowed to access locomotor activity and exploratory behavior of the tested rats.

### Localized ^1^H Magnetic Resonance Spectroscopy (MRS)

Rats were anesthetized with 2% isoflurane (Attane, Minrad, USA) in oxygen (PanGas, Ecublens, Switzerland), and then intubated and ventilated with a pressure-driven ventilator (MRI-1, CWE incorporated, PA, USA). Catheters were placed into the femoral artery for monitoring blood gases, glucose and blood pressure, and into the femoral vein for infusion of saline solutions containing α-chloralose (Acros Organics, Geel, Belgium) or D-glucose (Sigma-Aldrich, Switzerland). Rats were placed in a home-built holder that ensures a fixed and stable position of the skull for extended scanning times. Body temperature was maintained around 37.5°C with a warm water circulation system based on the feedback from a rectal temperature probe. Temperature, arterial blood pressure, heart rate, and respiratory rate were continuously monitored with an animal monitoring system (SA Instruments, NY, USA). Before inserting the animal in the bore of the magnet, anesthesia was switched to α-chloralose (intravenous bolus of 80 mg/kg, and continuous infusion of 25 mg/kg/h). D-glucose [20% (w/v) solution] was infused at a rate adjusted based on the concomitantly measured arterial plasma glucose concentrations to achieve stable targeted glycaemia levels. NMR measurements were performed after each glucose level had been stable for at least 15 min (Duarte and Gruetter, 2012). Arterial pH and pressures of O_2_ and CO_2_ were measured using a blood gas analyser (AVL Compact 3, Diamond Diagnostics, MA, USA). Concentration of glucose in arterial plasma samples was quantified by the glucose oxidase method, using a multi-assay analyser (GM7 Micro-Stat, Analox Instruments, UK).

All experiments were carried out as previously described (Duarte et al., 2009a) using a Varian INOVA spectrometer (Agilent Technologies, Palo Alto, CA, USA) interfaced to an actively-shielded 9.4 T magnet with a 31 cm horizontal bore (Magnex Scientific, Abingdon, UK), and a homebuilt 10 mm ^1^H quadrature surface coil. The rat brain was positioned in the isocentre of the magnet and located with fast-spin-echo images with 5 s repetition time, effective echo time of 52 ms and echo train length of 8. Shimming was performed with FAST(EST)MAP (Gruetter and Tkáč, 2000), and ^1^H NMR spectra were acquired from a volume of interest (VOI) of 18 μL placed in the left dorsal hippocampus using SPECIAL spectroscopy, with echo time of 2.8 ms and repetition time of 4 s (Mlynárik et al., 2006). Spectra were analyzed using LCModel (Stephen Provencher Inc., Oakville, Ontario, Canada), including a macromolecule spectrum in the database, as previously described (Mlynárik et al., 2006; Duarte et al., 2009a). The unsuppressed water signal measured from the same VOI was used as an internal reference (assuming the existence of 80% of water in the brain tissue) for the absolute quantification of the following metabolites: glucose (Glc), ascorbate (Asc), phosphoethanolamine (PE), creatine (Cr), phosphocreatine (PCr), *myo*-inositol (Ins), taurine (Tau), *N*-acetylaspartate (NAA), aspartate (Asp), glutamate (Glu), glutamine (Gln), γ-aminobutyrate (GABA), alanine (Ala), lactate (Lac), β-hydroxybutyrate (βHB), glycerophosphocholine (GPC), phosphocholine (PCho), glutathione (GSH), *N*-acetylaspartylglutamate (NAAG), *scyllo*-inositol (scyllo). The Cramér-Rao lower bound (CRLB) was provided by LCModel as a measure of the reliability of the apparent metabolite concentration quantification. CRLBs above 30% were systematically associated to *scyllo*-inositol, which was thus not used for further analyses. The remaining metabolites were quantified with CRLBs below 30%.

### Determination of Glucose Transport Kinetics

The determination of hippocampal glucose by MRS *in vivo* as function of plasma glucose was used to estimate kinetic parameters of glucose transport across the blood-brain-barrier (BBB). Steady-state brain glucose transport kinetics was modeled with a four-state conformational model that accounts for reversibility and trans-acceleration of the glucose carrier (Duarte et al., 2009b). Hippocampal glucose at steady-state was fitted to the following equation

$$\begin{array}{l} {\text{G}}_{\text{hipp}}={\text{V}}_{\text{d}}\frac{\left(\frac{{\text{T}}_{\text{max}}}{\text{CM}{\text{R}}_{\text{glc}}}-\text{1}\right){\text{G}}_{\text{p}}-{\text{K}}_{\text{t}}}{\frac{{\text{T}}_{\text{max}}}{\text{CM}{\text{R}}_{\text{glc}}}+\text{1}+\frac{{\text{G}}_{\text{p}}}{{\text{K}}_{\text{ii}}}} \end{array}$$

where G_hipp_ and G_p_ are the concentrations of glucose in the hippocampus (in μmol/g) and plasma (in mmol/L), respectively. CMR_glc_ is the cerebral metabolic rate of glucose. T_max_ denotes the apparent maximal transport rate across the BBB (μmol/g/min), K_t_ and K_ii_ denote the apparent Michaelis and iso-inhibition constants (in mmol/L), *V*_d_ = 0.77 mL/g is the volume of the physical distribution space of glucose in the hippocampus (see Duarte et al., 2009b for details).

### Western Blot Analysis

Immediately after the MRS experiment, rats were decapitated, the brain was rapidly removed, and the hippocampus dissected. Whole membranes and synaptosomes (i.e., synaptic-enriched) membranes were prepared (Rebola et al., 2005; Cunha et al., 2006), and Western blot analysis of proteins in these hippocampal membrane preparations was performed using previously detailed methods (Duarte et al., 2007; Kaster et al., 2015). Western blot analysis of A_2A_R was carried out as detailed by Hurtado-Alvarado et al. (2016), using the avidin-biotin Vectastain Elite kit (Vector Laboratories, Burlingame, CA-USA) for immunoreactivity amplification. The primary antibodies against the synaptic protein synaptosome-associated protein of 25 kDa (SNAP25; from Sigma, Sintra, Portugal), and against the glial fibrillary acidic protein (GFAP; from Sigma) were used at a dilution of 1:5,000. Antibodies against synaptophysin, α-tubulin and β-actin were purchased from Sigma and used at 1:10,000. Anti-postsynaptic density protein of 95 kDa (PSD95; from Chemicon) was used at 1:20,000; anti-vimentin (Sigma) and anti-microtubule-associated protein type 2 (MAP2; from Santa Cruz Biotechnology, Santa Cruz, CA, USA) at 1:1,000. Antibodies against A_1_R (Affinity Bioreagents, Golden, CO-USA) and A_2A_R (Abcam, Cambridge, UK) were used at a dilution of 1:600.

### Electrophysiological Recordings

Electrophysiological recordings of synaptic transmission and plasticity were performed in superfused hippocampal slices, as previously described (Costenla et al., 2011; Kaster et al., 2015; Silva et al., 2018). Briefly, a rat was deeply anesthetized with 2-bromo-2-chloro-1,1,1-trifluoroethane (halothane; no reaction to handling or tail pinch, while still breathing) before decapitation. The brain was rapidly removed and cooled in an artificial cerebrospinal fluid (aCSF) solution containing (in mmol/L): 124 NaCl, 3 KCl, 2 CaCl_2_, 1 MgCl_2_, 1.25 NaH_2_PO_4_, 10 glucose, 26 NaHCO_3_, pH 7.4; 290–310 mOsm, gassed with 95% O_2_, and 5% CO_2_. Coronal hippocampal slices (400 μm thick) were prepared with a manual Vibratome 1,500 sectioning system (Vibratome, Germany), and allowed to recover for 1 h at room temperature in a Harvard Apparatus resting chamber filled with gassed aCSF. Individual dorsomedial hippocampal slices were transferred to a submerged recording chamber and continuously superfused at a rate of 4 mL/min with gassed aCSF kept at 30.5°C. A bipolar concentric stimulation electrode (SNE-100; Kopf, Germany) was placed over the Schaffer fibers delivering rectangular pulses (550 μA) of 0.1 ms duration applied with a Digitimer DS3 stimulator (Digitimer LTD, UK) once every 20 s. The evoked field excitatory postsynaptic potentials (fEPSPs) were recorded through an extracellular borosilicate microelectrode filled with 4 mol/L NaCl (2–5 MΩ resistance) placed in the *stratum radiatum* of the CA1 area, coupled to an ISO-80 amplifier (World Precision Instruments, Hitchin, UK). Averages of four consecutive responses acquired with a 1 kHz cut-off were digitalized and continuously monitored on a personal computer with the WINLTP 1.1 program (Anderson and Collingridge, 2001) to quantify the initial slope of the averaged fEPSPs, used to estimate the effect of drugs, added to the superfusion solution.

After obtaining a stable baseline, we first carried out an input/output curve to select a stimulus intensity triggering 40–50% of the maximal amplitude. We then tested the effects of 2-chloroadenosine (CADO, the closest and chemically stable analog of adenosine; from Tocris, Bristol, UK) and 1,3-dipropyl-8-cyclopentylxantine (DPCPX, a selective antagonist of adenosine A_1_R; from Tocris) on basal synaptic transmission. Alternatively, we tested the effect of 2-(2-furanyl)-7-(2-phenylethyl)-7H-pyrazolo[4,3-e][1,2,4]triazolo[1,5-c]pyrimidin-5-amine (SCH58261, a selective antagonist of adenosine A_2A_R; from Sigma) on long-term potentiation (LTP). LTP was induced with a high-frequency train (100 Hz for 1 s) and was quantified as the percentage change between the fEPSP slopes 60 min after and 15 min before the train.

### Statistics

Data was analyzed using ANOVA with two factors (diabetes and caffeine treatment). For the metabolic profile analysis, all metabolite concentrations were analyzed together with a multivariate ANOVA. Significant differences were considered for *P* < 0.05. Multiple comparisons after ANOVA were performed with Fisher's least significant difference (LSD) tests upon significant diabetes effect or diabetes-caffeine interaction. Two-tailed Student *t*-tests were used to compare caffeine intake and caffeine serum concentration between GK and Wistar rats, as well as the effects of CADO and DPCPX on synaptic transmission. Results are reported as mean ± SEM unless otherwise stated.

## Results

**Figure 1:**
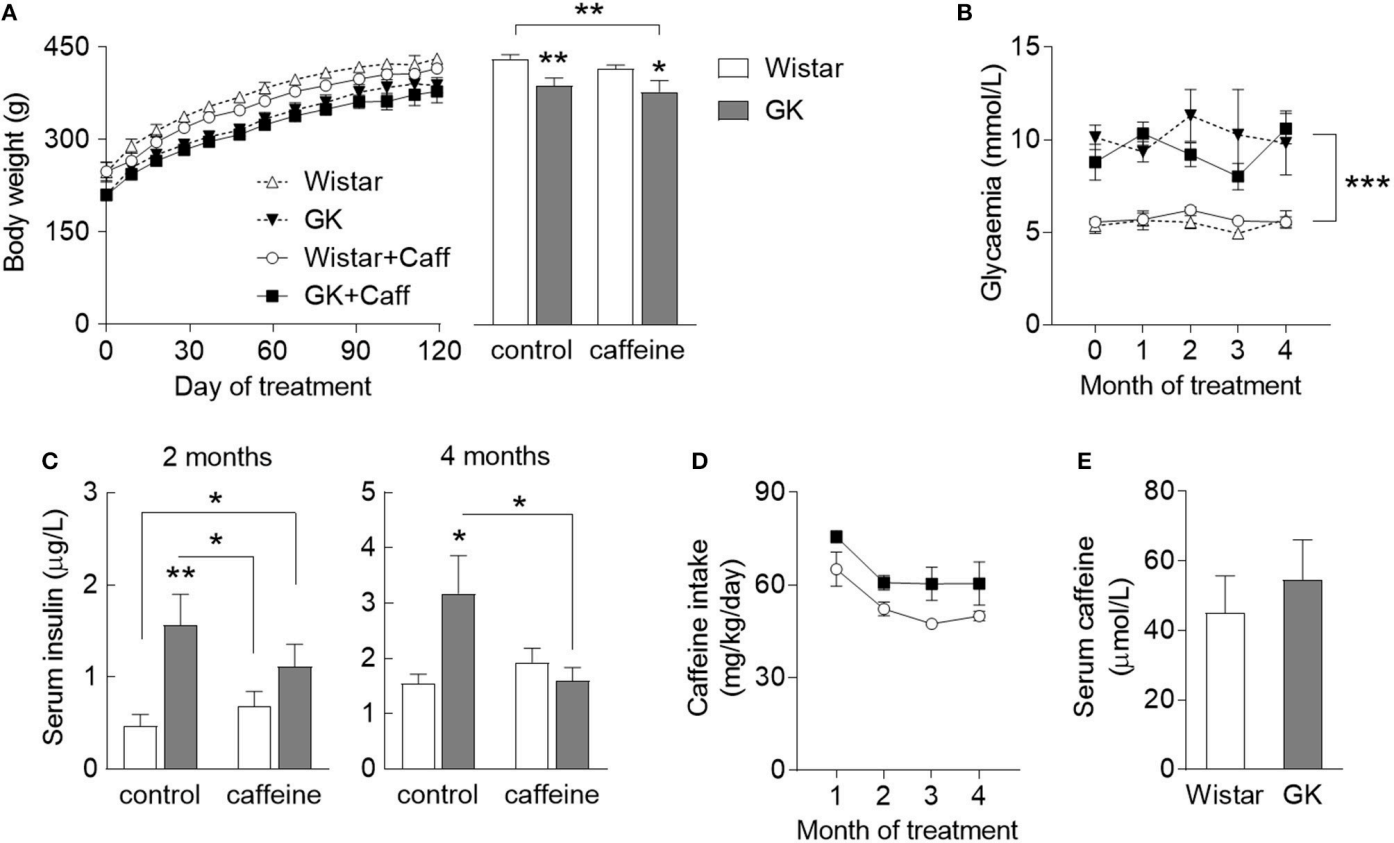
Characteristics of diabetic GK rats (filed symbols/bars) and control Wistar rats (open symbols/bars), namely body weight **(A)** across the caffeine treatment period (line graph) and at the end of the study (bar graph), glycaemia **(B)**, concentration in the serum insulin determined after 2 and 4 months of treatment **(C)**, caffeine intake measured across the treatment period **(D)**, and concentration of serum caffeine after 4 months of caffeine exposure **(E)**. Caffeine (1 g/L) was provided through the drinking water for 4 months, starting at 2 months of age. Data are mean ± SEM of 8 rats per group. Symbols represent LSD test results after ANOVA with either significant diabetes effect or significant diabetes-caffeine interaction: ^*^P < 0.05, ^**^P < 0.01, ^***^P < 0.001 for GK vs. Wistar in the respective treatment group or as indicated.

To test the role of caffeine consumption in the prevention of diabetes-induced hippocampal alterations, GK rats and age-matched controls were allowed to consume caffeine for 4 months, starting at 2 months of age. During the period when the rats had free access to 1 g/L caffeine solution, body weight, and pre-prandial glycaemia were monitored and insulin plasma levels were quantified 2 months after starting caffeine intake and at the end of the experiment. GK rats were smaller than controls independent of caffeine consumption, which had no significant effect on body weight (diabetes *P* = 0.002, caffeine *P* = 0.275, interaction *P* = 0.794; Figure 1A). T2D had a significant effect on fed glycaemia (*P* < 0.001), which was not impacted by caffeine treatment (caffeine *P* = 0.779, interaction *P* = 0.935; Figure 1B). Relative to controls, GK rats showed an increase in serum insulin concentration after 2 and 4 months of treatment (*P* = 0.002 and *P* = 0.036, respectively). At 4 months of treatment, caffeine prevented the diabetes-associated hyperinsulinemia (caffeine *P* = 0.212, diabetes *P* = 0.240, interaction *P* = 0.050; Figure 1C). Caffeine intake was slightly lower in Wistar than GK rats, but not significantly different (*P* = 0.063; Figure 1D). Serum levels of caffeine at the end of the treatment period were similar in diabetic and control rats (*P* = 0.558; Figure 1E).

### Caffeine Consumption Prevents Spatial Memory Impairment in GK Rats

**Figure 2:**
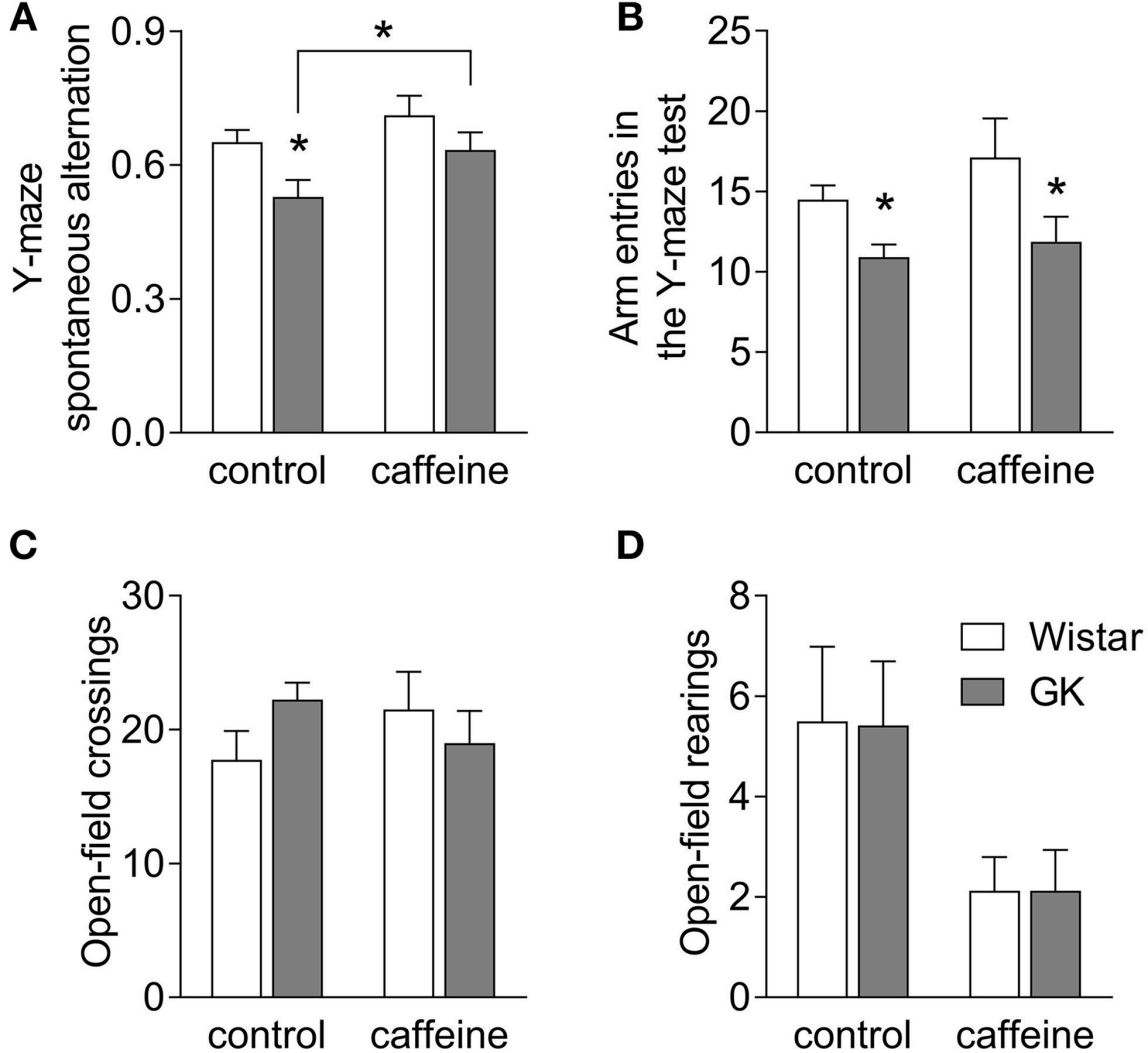
Caffeine consumption improves spatial working memory deficits in diabetic GK rats. GK rats displayed reduced spontaneous alternation in the Y-maze, when compared to controls, but not if treated with caffeine for 4 months **(A)**. The number of entries in the Y-maze arms was lower in GK compared to control rats irrespective of caffeine consumption **(B)**. The number of crossings in the open-field arena was unaltered by diabetes or caffeine consumption **(C)**, but the number of rearing events was reduced upon chronic caffeine consumption **(D)**. Data are mean ± SEM of 8 rats per group. Symbols represent LSD test results after ANOVA with either significant diabetes effect or significant diabetes-caffeine interaction: ^*^P < 0.05 for GK vs. Wistar in the respective treatment group or as indicated.

Hippocampal-dependent spatial memory was tested in a Y-maze 2 days before MRS *in vivo* at 6 months of age, i.e., after 4 months of caffeine exposure. Both diabetes and caffeine treatment affected spatial memory performance in the Y-maze (diabetes *P* = 0.011, caffeine *P* = 0.033, interaction *P* = 0.549). *Post-hoc* testing revealed that diabetes in GK rats caused a reduction of the spontaneous alternation in the Y-maze task when compared to controls (−19 ± 3%; *P* < 0.001; Figure 2A), which was ameliorated by 4 months of caffeine consumption. GK rats also showed a significant reduction in the number of entries in the Y-maze arms, independently of caffeine intake (diabetes *P* = 0.003, caffeine *P* = 0.199, interaction *P* = 0.546; Figure 2B). Nevertheless, diabetes was not associated with exploratory or locomotor impairment as gauged by similar exploration of the open-field arena (Figures 2C,D). Interestingly, caffeine impacted the number of rearing events in the open-field test (caffeine *P* = 0.014, diabetes *P* = 0.974, interaction *P* = 0.974), without impacting the number of crossing events between quadrants of the arena (caffeine *P* = 0.908, diabetes *P* = 0.465, interaction *P* = 0.113).

### Diabetes-induced Metabolic Alterations

**Figure 3:**
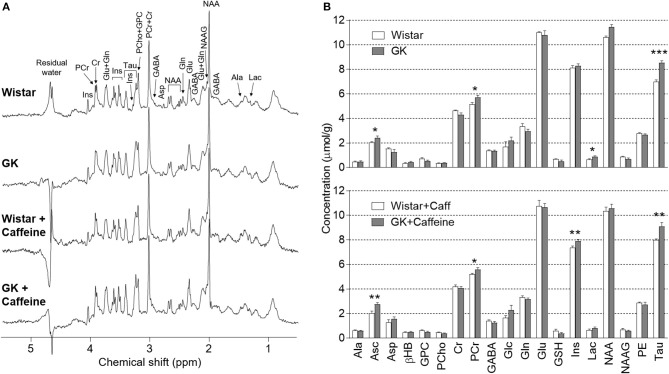
Diabetes-induced metabolic alterations persisted upon caffeine treatment. **(A)** shows representative ^1^H spectra acquired *in vivo* at 9.4 T from the dorsal hippocampus rats under normoglycaemia. An increase of taurine resonances at 3.25 and 3.42 ppm in the spectra from GK rats is clearly visible. The spectra were measured by the SPECIAL sequence with echo time of 2.8 ms, repetition time of 4 s, 640 scans and in a volume of 18 μL. For resolution enhancement, a shifted Gaussian function (gf = 0.12 and gsf = 0.05) was applied before Fourrier transformation. Zero-phase but not baseline was corrected. **(B)** shows mean metabolite concentrations in the hippocampus of Wistar (*n* = 6), GK (*n* = 8), caffeine-treated Wistar (*n* = 7) and caffeine-treated GK (*n* = 8) rats, at plasma glucose below 8 mmol/L. None of the metabolite concentrations showed caffeine-diabetes interaction. Results from *post-hoc* tests on metabolite levels after significant diabetes effect or caffeine-diabetes interaction are represented by ^*^P < 0.05, ^**^P < 0.01, and ^***^P < 0.001 for GK vs. Wistar in the respective treatment group.

^1^H spectra were acquired at 9.4 Tesla from the dorsal hippocampus of rats with half-height linewidths of 8–14 Hz and signal-to-noise ratios above 20, as reported by the LCModel. By inspecting these representative spectra (Figure 3A), one notices increased resonances of taurine in the hippocampus of GK rats relative to controls. This was in fact the most prominent metabolic alteration present in this model of T2D (see statistics below). Nineteen metabolites were quantified under normoglycaemia for the four experimental groups (Figure 3B). Analysis with a multivariate ANOVA to the whole metabolic profile indicated significant effects of diabetes (*P* = 0.002) and caffeine (*P* = 0.028) without diabetes-caffeine interaction (*P* = 0.493), which suggests cumulative effects of both factors. Furthermore, none of the levels of metabolites showed significant interaction between diabetes and caffeine in follow-up individual analyses.

Diabetes affected the concentration of taurine (*P* < 0.001), ascorbate (*P* < 0.001), creatine (*P* = 0.002), phosphocreatine (*P* < 0.001), glutamine (*P* = 0.041), *myo*-inositol (*P* = 0.011), lactate (*P* = 0.005), and glycerophosphorylcholine (*P* < 0.001). In *post-hoc* analyses comparing GK and Wistar rats in the absence of caffeine, GK rats only displayed significant increases in the levels of taurine (+22 ± 3%, *P* < 0.001), ascorbate (+20 ± 9%, *P* = 0.038), lactate (+34 ± 14%, *P* = 0.035), and phosphocreatine (+11 ± 4%, *P* = 0.028). Moreover, we observed a tendency for reduced creatine levels in GK rats (−7 ± 4%, *P* = 0.080 vs. Wistar), which resulted in a significant increase of phosphocreatine-to-creatine ratio (PCr/Cr; diabetes *P* = 0.004, caffeine *P* = 0.150, interaction *P* = 0.378). High PCr/Cr under normoglycaemia suggests that the hippocampus of GK rats is metabolically adapted to the diabetic condition in order to provide sufficient energy for basal oxidative metabolism.

On top of the effect of diabetes, caffeine consumption for 4 months had an effect on the concentration of creatine (*P* = 0.027), *myo*-inositol (*P* < 0.001), *N*-acetylaspartylglutamate (*P* = 0.036) and taurine (*P* = 0.023). Notably, when compared to untreated controls, Wistar rats consuming caffeine exhibited reduced *myo*-inositol (−9 ± 2%, *P* = 0.002) and increased taurine (+15 ± 2%, *P* < 0.001) concentrations in the hippocampus. *Post-hoc* analyses within the caffeine-treated rats revealed higher levels of ascorbate (+35 ± 9%, *P* = 0.002), taurine (+14 ± 4%, *P* = 0.004), *myo*-inositol (7 ± 2%, *P* = 0.006), and phosphocreatine (7 ± 3%, *P* = 0.045) in GK than Wistar rats. Altogether, these results suggest that the caffeine-induced changes, namely in the osmolites *myo*-inositol and taurine, add to those induced by T2D.

### Hippocampal Glucose Homeostasis

**Figure 4:**
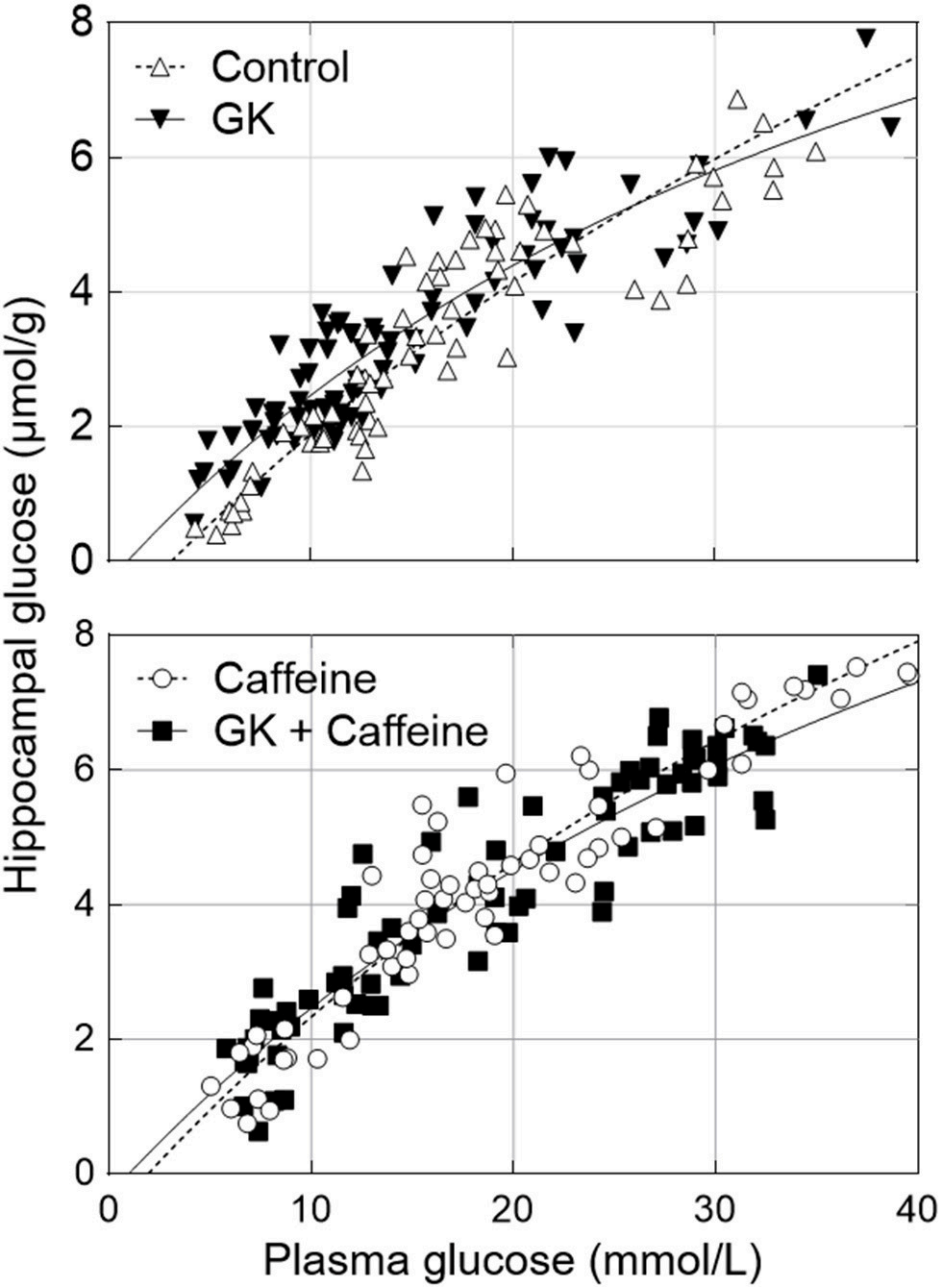
Relationship between hippocampal and plasma glucose concentrations in control and GK rats treated or not with caffeine (1 g/L). Data corresponds to steady-state hippocampal glucose levels determined from ^1^H NMR spectra measured during 40 min after plasma glucose was stable for at least 15 min. The kinetic parameters of glucose transport estimated from these datasets are presented in Table 2 and all measurements from *n* = 6–8 animals per group were pooled together.

We have recently reported that diabetes impairs global glucose transport and consumption in the brain, without changes in the brain to plasma glucose levels (Girault et al., 2018). Since the hippocampus is particularly affected by T2D in experimental models, we measured hippocampal glucose concentration at several steady-state plasma glucose levels to test whether BBB transport of glucose in this region remains sufficient to feed metabolism. Physiology parameters measured during the periods of MRS were similar in all four experimental groups (Table 1). Glucose concentration in the hippocampus was similar for GK and control rats, and was dependent on plasma glucose levels (Figure 4). This indicates that the relation between glucose transport and consumption is not altered in the hippocampus of insulin-resistant GK rats, confirming previous observations in the whole brain (Girault et al., 2018). Kinetic parameters for hippocampal glucose transport estimated with the four-state conformational model were similar across the four experimental groups (Table 2). Indeed, neither T2D nor habitual caffeine consumption affected T_max_/CMR_glc_, suggesting that glucose transport at the BBB in our experimental conditions matches the glucose consumption needs in the hippocampus.

**Table 1:** Mean physiologic parameters measured at 5 different intervals of steady-state plasma glucose concentration in MRS experiments.

**Plasma glucose range (mM)**		**<8**	**8–14**	**14–20**	**20–26**	**>26**
Body Temperature (°C)	Control	37.5 ± 0.2	37.4 ± 0.1	37.5 ± 0.1	37.2 ± 0.2	37.3 ± 0.1
	Caffeine	37.5 ± 0.2	37.2 ± 0.2	37.3 ± 0.1	37.1 ± 0.1	37.4 ± 0.1
	GK	37.0 ± 0.1	37.3 ± 0.2	37.2 ± 0.3	37.4 ± 0.2	37.0 ± 0.1
	GK + Caffeine	37.5 ± 0.1	37.3 ± 0.2	37.5 ± 0.2	37.6 ± 0.1	37.2 ± 0.1
Arterial pH	Control	7.34 ± 0.01	7.34 ± 0.01	7.34 ± 0.01	7.31 ± 0.02	7.33 ± 0.03
	Caffeine	7.42 ± 0.02	7.38 ± 0.01	7.35 ± 0.01	7.35 ± 0.01	7.33 ± 0.02
	GK	7.41 ± 0.02	7.40 ± 0.02	7.42 ± 0.02	7.40 ± 0.01	7.39 ± 0.01
	GK + Caffeine	7.44 ± 0.01	7.45 ± 0.01	7.39 ± 0.02	7.39 ± 0.02	7.38 ± 0.02
P_a_CO_2_ (mm Hg)	Control	44.7 ± 2.0	44.7 ± 1.6	46.8 ± 1.3	44.9 ± 1.9	42.1 ± 2.0
	Caffeine	39.9 ± 2.9	41.5 ± 2.7	45.7 ± 3.9	41.7 ± 2.7	41.4 ± 2.9
	GK	39.0 ± 3.4	38.6 ± 2.0	37.2 ± 2.7	40.3 ± 2.6	43.2 ± 2.3
	GK + Caffeine	35.7 ± 1.7	39.5 ± 4.7	38.9 ± 3.0	40.7 ± 3.0	40.1 ± 1.3

*Data is mean ± SEM of 6 to 8 rats in each experimental group*.

**Table 2:** Apparent Michaelis-Menten constant (K_t_), iso-inhibition constant (K_ii_) and ratio of maximal transport rate (T_max_) to cerebral glucose consumption rate (CMR_glc_) for the glucose transport across the BBB, estimated with the 4-state conformational model from the relationship between hippocampal and plasma glucose concentrations in control, GK, caffeine-treated control, caffeine-treated GK rats (data in Figure 4).

	**T_**max**_/CMR_**glc**_**	**K_**t**_**	**K_**ii**_**
Control	2.3 (1.9–3.1)	4.2 (2.4–7.4)	22.9 (8.4–161.8)
GK	2.5 (2.0–4.7)	1.5 (0.0–8.2)	12.9 (3.3–55.0)
Caffeine-treated control	2.5 (1.9–4.3)	2.9 (0.1–10.4)	19.1 (5.0–201.9)
Caffeine-treated GK	2.4 (1.9–6.8)	1.4 (0.0–17.4)	16.2 (2.3–74.9)

*Values are mean (95% confidence interval). Units of K_t_ and K_ii_ are mmol/L, T_max_/CMR_glc_ is adimensional*.

### Synaptic Alterations in the Hippocampus of GK Rats

**Figure 5:**
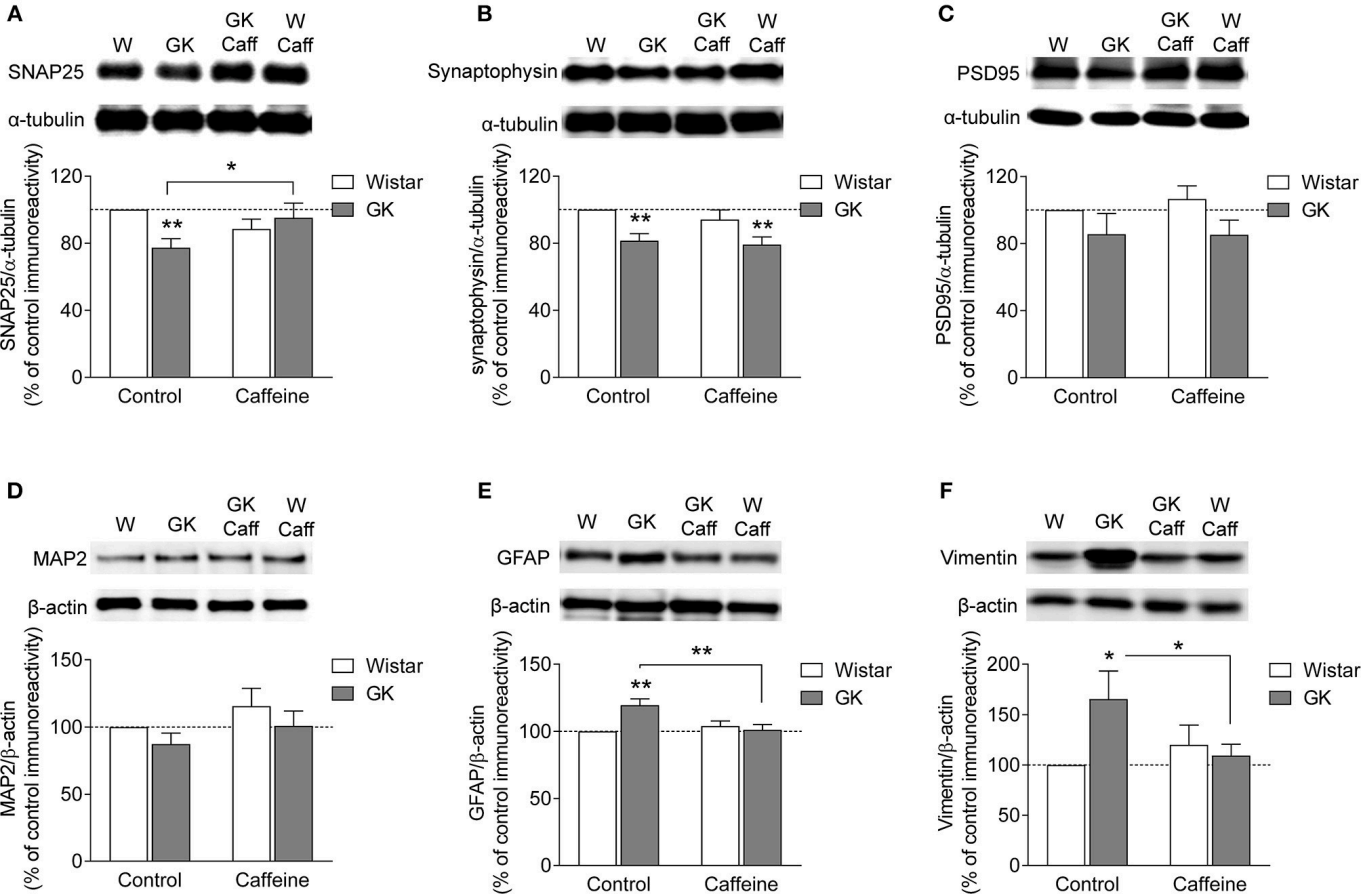
Caffeine consumption attenuated diabetes-induced synaptic degeneration and prevented astrogliosis in the hippocampus of GK rats. Western blot analysis revealed that hippocampal synaptosomal membranes from diabetic GK rats displayed reduced immunoreactivity of both SNAP25 **(A)** and synaptophysin **(B)** but not PSD95 **(C)**, when compared to Wistar rats (W). In total hippocampal membranes, diabetes failed to alter MAP2 immunoreactivity relative to controls **(D)**. Caffeine (Caff) consumption prevented diabetes-induced reduction of SNAP25 but not synaptophysin. Western blot analysis revealed that both GFAP **(E)** and vimentin **(F)** immunoreactivity was increased in total hippocampal membranes from GK compared to control rats, an effect prevented by the consumption of caffeine. Samples were loaded in the SDS-PAGE gel at a protein concentration of 25 μg. Immunoreactivity of proteins of interest was normalized to either α-tubulin or β-actin, and expressed as percentage of control in the same Western blot experiment. In the graphs, open and filled bars represent Wistar and GK rats, respectively. Data are mean±SEM of *n* = 5–8 animals per experimental group. Symbols represent LSD test results after ANOVA with either significant diabetes effect or significant diabetes-caffeine interaction: ^*^P < 0.05, ^**^P < 0.01 for GK vs. Wistar in the respective treatment group or as indicated.

The putative degeneration of synapses was evaluated by quantifying the density of two presynaptic proteins in nerve terminal-enriched membrane preparations. As shown to occur in streptozotocin-induced diabetic rats (Duarte et al., 2006, 2009a) and NONcNZO10/Ltj diabetic mice (Duarte et al., 2012), the hippocampus of GK rats displayed synaptic degeneration, as suggested by reduced immunoreactivity of SNAP25 (−23 ± 5%, *P* = 0.009, *n* = 8, Figure 5A) and synaptophysin (−19 ± 3%, *P* = 0.007, *n* = 5, Figure 5B), when compared to control rats. Chronic caffeine consumption for 4 months did not significantly affect the immunoreactivity of these synaptic markers, whereas it prevented the T2D-induced reduction of SNAP25 (caffeine *P* = 0.587, diabetes *P* = 0.176, interaction *P* = 0.0162; Figure 5A) but not synaptophysin immunoreactivity (caffeine *P* = 0.349, diabetes *P* = 0.001, interaction *P* = 0.681; Figure 5B). To evaluate whether T2D also affected the post-synaptic compartment, we quantified the immunoreactivity of post-synaptic density-95 (PSD95), a prototypical postsynaptic marker. The immunoreactivity of PSD95 was not significantly altered in synaptic membranes of GK rats when compared to controls in the absence or presence of caffeine treatment (caffeine *P* = 0.719, diabetes *P* = 0.053, interaction *P* = 0.692; Figure 5C). Furthermore, total membranes from the hippocampus of GK and control rats also displayed similar MAP2 immunoreactivity (caffeine *P* = 0.129, diabetes *P* = 0.154, interaction *P* = 0.915; Figure 5D). Altogether these results suggest a main T2D-induced defect at presynaptic level.

### Caffeine Consumption Prevents Diabetes-Induced Astrogliosis

Astrogliosis has been reported in several neurodegenerative diseases, including diabetes (e.g., Saravia et al., 2002; Baydas et al., 2003; Duarte et al., 2009a, 2012). This was now also found in the hippocampus of GK diabetic rats. Thus, when compared to controls, total hippocampal membranes prepared from GK rats exhibited increased immunoreactivity of GFAP (+20 ± 5%, *P* < 0.001, *n* = 8; Figure 5E) and vimentin (+65 ± 28%, *P* = 0.010, *n* = 5, Figure 5F). While caffeine intake was devoid of significant effects on the measured glial proteins in control Wistar rats, it prevented T2D-induced increase in the immunoreactivity of both astroglial-specific proteins GFAP (caffeine *P* = 0.053, diabetes *P* = 0.026, interaction *P* = 0.003, Figure 5E) and vimentin (caffeine *P* = 0.280, diabetes *P* = 0.108, interaction *P* = 0.030, Figure 5F).

### Altered Density of Adenosine A_1_ and A_2A_ Receptors in the Hippocampus

**Figure 6:**
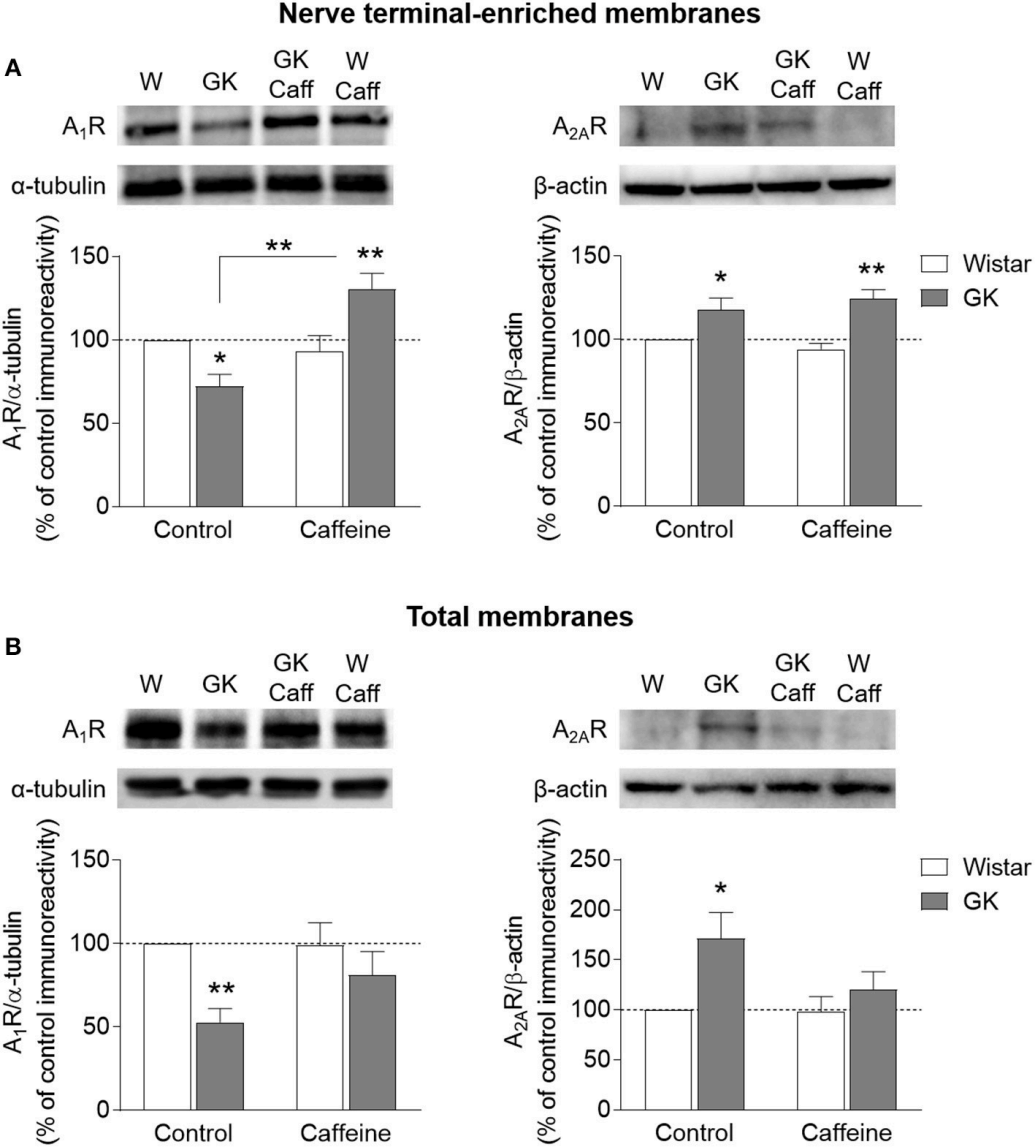
Density A_1_R and A_2A_R analyzed by Western blot in nerve terminal-enriched membranes **(A)** and total membranes **(B)** from the hippocampus of GK and Wistar (W) rats, either receiving 1 g/L caffeine (Caff) or tap water. Samples were loaded in SDS-PAGE gels at a protein concentration of 40 or 150 μg for A_1_R (*n* = 6–8) and A_2A_R (*n* = 2–3), respectively. Immunoreactivity was normalized to either α-tubulin or β-actin, and calculated as percentage of control in the same Western blot experiment. Symbols represent LSD test results after ANOVA with either significant diabetes effect or significant diabetes-caffeine interaction: ^*^P < 0.05, ^**^P < 0.01 for GK vs. Wistar in the respective treatment group or as indicated.

The density of adenosine receptors was evaluated by Western Blot in whole membranes and nerve terminal-enriched membranes prepared from the hippocampus. In synaptosomes, there was a significant interaction between effects of diabetes and caffeine on the density of A_1_R (interaction *P* < 0.001, diabetes *P* = 0.539, caffeine *P* = 0.002, Figure 6A). When compared to controls, *post-hoc* analyses revealed a significant reduction of A_1_R immunoreactivity in GK rats (-28 ± 7%, *P* = 0.014, *n* = 8), which was reversed upon caffeine consumption (+40 ± 10%, *P* = 0.002, *n* = 8). In contrast, diabetes caused a significant increase in levels of A_2A_R in synaptic membranes, independently of caffeine consumption (diabetes *P* < 0.001, caffeine *P* = 0.919, interaction *P* = 0.223; Figure 6A). In the absence of caffeine, hippocampal synaptosomes from GK rats showed a 18±7% increase of A_2A_R immunoreactivity (*P* = 0.031, *n* = 3). Within caffeine treated rats, there was a T2D-induced increase of A_2A_R immunoreactivity of 32±6% (*P* = 0.014, *n* = 3). In total membranes, T2D was associated to a major reduction of A_1_R levels (diabetes *P* = 0.006, caffeine *P* = 0.210, interaction *P* = 0.181), which was significantly different from controls only in the absence of caffeine treatment (−47 ± 8%, *P* = 0.005, *n* = 6; Figure 6B). In turn, the opposite effect was observed for A_2A_R immunoreactivity, which increased in GK rats compared to controls in the absence (+71 ± 26%, *P* = 0.046, *n* = 2) but not in the presence of caffeine treatment (diabetes *P* = 0.057, caffeine *P* = 0.205, interaction *P* = 0.232). It should be noted however that the detection of changes on A_2A_R density by Western blot suffered from technical challenges due to the known low immunoreactivity signal from the hippocampus of 6 month old rats (e.g., Rebola et al., 2003; Canas et al., 2009a). This is especially critical in total membranes from the rat hippocampus, in which the density of A_2A_R is about half of that in synaptosomal membranes (e.g., Rebola et al., 2005; Duarte et al., 2006). Therefore, the present A_2A_R density changes should be interpreted in a qualitative rather than quantitative manner.

### Altered Efficiency of Adenosine Receptors Controlling Hippocampal Synaptic Transmission and Plasticity

**Figure 7:**
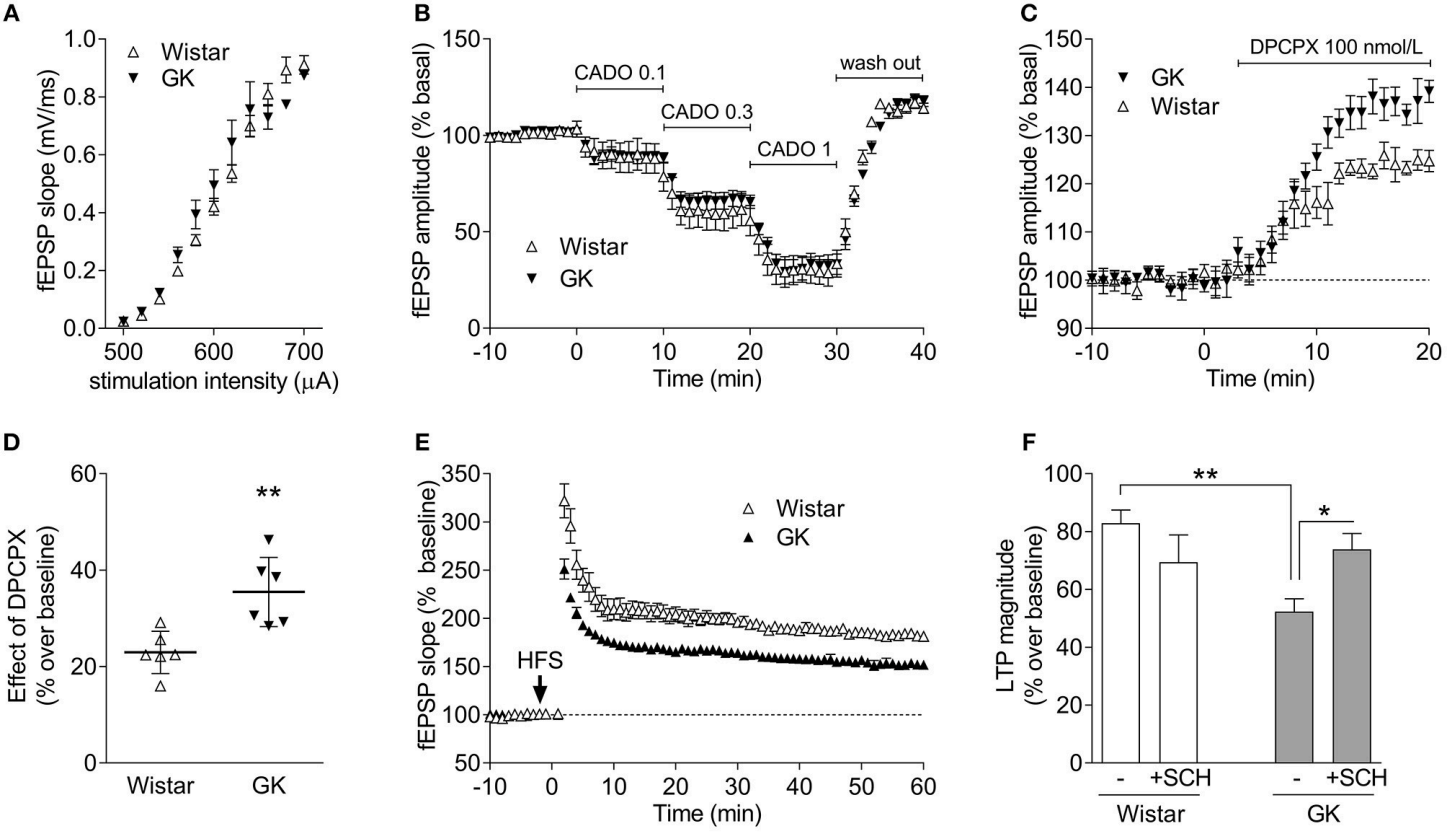
GK rats display an unchanged A_1_R-mediated inhibition of excitatory synaptic transmission in the hippocampus but an increased A_2A_R-mediated control of synaptic plasticity. **(A)** Superimposed input-output curves in GK (filed symbols) and Wistar rats (open symbols), indicating a similar density of excitatory innervation in Schaffer fiber-CA1 pyramid synapses of hippocampal slices. **(B)** Superimposable concentration-dependent curves of inhibition of CA1 hippocampal field excitatory post-synaptic potentials (fEPSPs) by the closest chemical analog of adenosine, 2-chloroadenosine (CADO; applied at concentrations of 0.1, 0.3, and 1 μmol/L), showing that the A_1_R-mediated inhibition of synaptic transmission is similar in GK and Wistar rats. The fEPSP slope before CADO application is normalized to 100%, and 0% corresponds to a complete inhibition of fEPSPs. **(C,D)** GK rats seem to have higher levels of endogenous extracellular adenosine tonically activating inhibitory A_1_R, as revealed by the larger disinhibition of fEPSPs by the selective A_1_R antagonist, DPCPX. **(E)** Averaged time course changes of fEPSP slope induced by a high-frequency stimulation train (HFS 100 Hz, 1 s, applied when indicated by the arrow) in hippocampal slices taken from Wistar (open symbols) or GK rats (filed symbols), showing that the amplitude of LTP is larger in Wistar than in GK rats. **(F)** The presence of the A_2A_R antagonist SCH58261 (50 nM, +SHC), which was applied 30 min before LTP induction and remained in the bath up to the end of the experiment, has a discrete effect in Wistar rats (open bars) and efficiently restores LTP amplitude back to control levels in GK rats (gray bars). The electrophysiological data are mean ± SEM of 5–6 animals per experimental condition. ^*^P < 0.05, ^**^P < 0.01 using either an unpaired Student's *t* test in **(C)** or a two-way ANOVA followed by a LSD *post-hoc* test in **(F)**.

The near superimposable input/output curves obtained in hippocampal slices from Wistar and GK rats ascertains that there were no changes in the density of excitatory inputs in Schaffer fibers CA1 pyramid synapses (Figure 7A), enabling a direct comparison of the efficiency of A_1_R and A_2A_R to control synaptic transmission and plasticity. Thus, we tested the ability of A_1_R to control basal synaptic transmission (Costenla et al., 2011) and found that the closest chemical analog of adenosine, 2-chloroadenosine (CADO), triggered a similar concentration-dependent inhibition of synaptic transmission (Figure 7B). In fact, the estimated EC_50_ of CADO to inhibit synaptic transmission was 0.56 μmol/L (95% confidence interval: 0.08–1.04 μmol/L, *n* = 6) in slices from Wistar rats, which was similar (*P* = 0.833) to the EC_50_ values obtained in slices from GK rats (0.61 μmol/L, 95% confidence interval: 0.23–1.00 μmol/L, *n* = 6). We then investigated if there were changes in the levels of endogenous adenosine tonically controlling basal excitatory transmission (Costenla et al., 2011). A supra-maximal but selective concentration (100 nmol/L) of the A_1_R antagonist DPCPX (Sebastião et al., 2000) caused a greater disinhibition of hippocampal synaptic transmission in GK rats compared to Wistar rats (*P* = 0.006; *n* = 6; Figures 7C,D). This suggests a preserved efficiency of A_1_R-mediated inhibition of synaptic transmission and higher levels of endogenous extracellular adenosine controlling synaptic transmission in GK rats.

We next compared synaptic plasticity in hippocampal slices from Wistar and GK rats to gauge the efficiency of A_2A_R that selectively control hippocampal synaptic plasticity (Rebola et al., 2008; Costenla et al., 2011). The amplitude of long-term potentiation (LTP) was lower (*P* = 0.0004, Figure 7E) in GK rats (52 ± 4% over baseline, *n* = 6) than in Wistar rats (83 ± 2% over baseline, *n* = 5). The selective A_2A_R antagonist SCH58261, used in a supramaximal and selective concentration of 50 nmol/L (Lopes et al., 2004), caused a discrete inhibition of LTP amplitude in Wistar rats (70 ± 9% over baseline, *n* = 5) and recovered the depressed LTP amplitude in GK rats to near control values (74 ± 5% over baseline, *n* = 6). A two-way ANOVA on LTP amplitude results showed a significant effect of diabetes [*F*_(1, 18)_ = 5.0; *P* = 0.038], no effect of applied drug [*F*_(1, 18)_ = 0.5; *P* = 0.496], and a significant effect of their interaction [*F*_(1, 18)_ = 9.0; *P* = 0.008]. *Post-hoc* analyses confirmed that LTP amplitude was decreased in GK compared to Wistar rats (*P* = 0.002), whereas SCH58261 recovered plasticity in GK rats (*P* = 0.014) but had a negligible effect in Wistar rats (*P* = 0.136).

## Discussion

The present study deepens our knowledge of the impact of T2D on cognitive function, which is not yet fully understood (Frisardi et al., 2010; Steculorum et al., 2014; Duarte, 2015). Diabetic rats displayed impaired hippocampal-dependent spatial memory, as suggested by reduced Y-maze spontaneous alternation. This diabetes-induced memory impairment was not accompanied by a modification of glucose transport to consumption ratio, that is, there was no alteration of hippocampal glucose concentration at a given glycaemia. Instead, when compared to controls, GK rats displayed alterations of the metabolic profile, synaptic dysfunction, and astrogliosis in the hippocampus. The causal relation between synaptic damage and astrogliosis and the memory impairment in GK rats is further emphasized by the observation that caffeine consumption had simultaneous beneficial effects on diabetes-induced spatial memory dysfunction, synaptic damage, and astrogliosis. We further observed that both caffeine and T2D have an impact on metabolism in the hippocampus. However, caffeine consumption did not prevent diabetes-induced metabolic changes. It remains to be ascertained whether caffeine-associated metabolic changes are beneficial for the diabetic brain.

Brain function relies on glucose as main source of energy, and resting brain glucose uptake and consumption are largely independent from circulating insulin (Hasselbalch et al., 1999). Although diabetes leads to inadequate glucose transport in peripheral tissues, brain glucose utilization may eventually adapt to a new metabolic condition upon diabetes (Pelligrino et al., 1992). Some studies reported a lack of effect of type 1 diabetes on the transport of glucose into the brain (Kainulainen et al., 1993; Simpson et al., 1999). Likewise, previous studies in humans reported that poorly controlled diabetes did not affect glucose transport into the brain (Fanelli et al., 1998). We have previously found that insulin-dependent rats have hippocampus to plasma glucose concentrations similar to controls (Duarte et al., 2009a). In GK rats, we recently demonstrated that T2D is associated to reduced glucose transport and consumption rates in the whole brain, without modifying brain to plasma glucose relationship at steady-state. The present results confirmed this observation in the dorsal hippocampus, which controls learning and memory. Unaltered glucose transport to consumption ratio (T_max_/CMR_glc_) implies an increased glucose concentration in the hippocampus under sustained hyperglycaemia. The high glucose level in the hippocampus may trigger osmolarity alterations and thus induce metabolic adaptation. This is expected to translate into a modified neurochemical profile, as was observed in the hippocampus of GK rats compared to controls under normoglycaemia (Figure 3). The most prominent alteration was an increase in the hippocampal concentration of the osmolite taurine. Surprisingly, the concentration of *myo*-inositol (another osmolite) was not substantially altered in the hippocampus of GK rats in the absence of caffeine, in contrast to what was observed in streptozotocin-induced diabetic rats (Duarte et al., 2009a) or Zucker diabetic obese rats (van der Graaf et al., 2004). However, these models of diabetes are characterized by sustained hyperglycaemia ranging from 25 to 30 mM of plasma glucose (Wilkes et al., 2005; Duarte et al., 2009a), while GK rats are subjected to a rather mild hyperglycaemia state (below 15 mmol/L). This tentatively suggests that *myo*-inositol levels may only increase upon more extreme hyperosmolarity.

T2D was also associated with increased levels of ascorbate in the hippocampus, which is in line with stimulation of ascorbate production in the rat liver under mild hyperglycaemia (Küstermann et al., 1998). Ascorbate is involved in the regulation of brain glycolysis and pentose phosphate pathway, as well as astrocyte-neuron metabolic interactions (Cisternas et al., 2014), and changes of its concentration in the hippocampus may be related with T2D-induced adaptations of energy metabolism (Girault et al., 2018). The non-deleterious but rather adaptive nature of these diabetic-induced metabolic changes in hippocampal metabolic profile is supported by the observation that GK rats at euglycaemia displayed augmented phosphocreatine-to-creatine ratio, compared to controls.

As previously observed in the hippocampus of insulin-dependent diabetic rats, caffeine consumption lowered *myo*-inositol concentration, and increased hippocampal levels of taurine. Taurine is an amino acid that, although present at 1 μmol/g in the human brain, it reaches relatively large concentrations in the rodent brain (above 5 μmol/g in rats and above 8 μmol/g in mice; Duarte, 2016), playing a major role as osmolyte (Duarte et al., 2009a, and references therein). Indeed, caffeine is able to control osmotic swelling via adenosine receptors (Wurm et al., 2008). In addition, taurine acts as an agonist at receptors of the GABAergic and glycinergic neurotransmitter systems (Albrecht and Schousboe, 2005), and caffeine controls taurine release from both neurons and glia via adenosine receptors (Hada et al., 1998). Taurine is transported into the mitochondrial matrix where it buffers pH to the optimal value for isocitrate dehydrogenase, which is a key enzyme of the tricarboxylic acid cycle regulating metabolism and oxidative phosphorylation, contributes to stabilize the pH gradient across the inner-membrane, and thus helps preserving mitochondrial function and preventing oxidative damage (Hansen et al., 2010). Therefore, this caffeine-associated increase of taurine levels in the diabetic hippocampus is likely related to neuroprotective functions.

Finally, it should be stressed that these adaptive metabolic modifications in the hippocampus of GK rats indeed seem to be caused by hyperglycaemia rather than by hyperinsulinemia since chronic consumption of caffeine prevented the later but not the former, and failed to prevent hippocampal metabolite alterations in GK rats, despite caffeine-induced metabolic changes.

The evaluation of hippocampal metabolite concentrations in Wistar and GK rats showed that this brain structure faces high glucose levels in diabetes at their fed glycaemia, which may lead to neurotoxicity and cellular damage. The present results indicate that T2D in GK rats caused neurodegeneration that does not affect the entire neuron, as suggested by unaltered MAP2 immunoreactivity, but instead occurs selectively at the presynaptic component of the nerve terminal, as previously proposed (Duarte et al., 2006, 2009a, 2012; Gaspar et al., 2010). In fact, GK rats displayed a reduced density of the presynaptic proteins SNAP25 and synaptophysin in the hippocampus, whereas the density of the postsynaptic protein PSD95 was not significantly altered relative to controls. The alteration of these presynaptic markers allowed sustaining synaptic transmission but was associated with an alteration of synaptic plasticity typified by a reduced amplitude of long-term potentiation in the hippocampal CA1 area of GK compared to Wistar rats. These synaptic modifications may eventually underlie the memory impairment observed in GK rats, as proposed to occur in Alzheimer's disease-associated neurodegeneration (Selkoe, 2002; Coleman et al., 2004). Together with synaptic dysfunction, we further found increased immunoreactivity of the glial-specific proteins GFAP and vimentin in the hippocampus of GK rats. This is in accordance with the occurrence of astrocytosis in the hippocampus, which was observed in other animal models of diabetes (e.g., Saravia et al., 2002; Baydas et al., 2003; Duarte et al., 2009a, 2012). Astrocytosis can be triggered by neuronal damage and contribute to further neuronal deterioration through the production of free radicals (e.g., Chao et al., 1996) and apoptotic factors (e.g., Crutcher et al., 1993; Fahnestock et al., 1996), leading to memory impairment (see Halassa and Haydon, 2010). In line with synaptic degeneration and astrocytosis, we have previously reported that GK rats show whole brain depression of neuronal oxidative metabolism and glutamate-glutamine cycle, and exacerbation of oxidative metabolism in astrocytes (Girault et al., 2018).

The relation between synaptotoxicity and astrogliosis with memory impairment in GK rats was further supported by the common ability of chronic caffeine consumption to simultaneously ameliorate or prevent these T2D-induced modifications. This is in agreement with the general neuroprotective action of chronic caffeine consumption against brain damage, which is largely mimicked by antagonists of adenosine A_2A_Rs (Cunha, 2005; Chen et al., 2007). In particular, both caffeine and selective A_2A_R antagonists are effective in improving memory performance upon noxious insults (Takahashi et al., 2008; Cunha, 2016), which was also observed in this study. Thus, neuroprotection and preservation of memory function by caffeine is likely associated with antagonism of A_2A_Rs at the synaptic level as well as in glial cells (Cunha, 2016). Notably, the over-functioning of A_2A_Rs is sufficient to impair memory performance (Li et al., 2015; Pagnussat et al., 2015). Accordingly, A_2A_Rs were up-regulated in the hippocampus of GK rats, similarly to what was observed in other animal models of T1D (Duarte et al., 2009a) or T2D (Duarte et al., 2012) and in a variety of conditions associated with memory dysfunction, such as aging (Rebola et al., 2003; Canas et al., 2009a; Temido-Ferreira et al., 2018) or Alzheimer's disease (Canas et al., 2009b; Espinosa et al., 2013; Viana da Silva et al., 2016; Silva et al., 2018). In fact, the only established molecular targets for caffeine at non-toxic concentrations, which were achieved in the present study, are adenosine receptors, mainly A_1_Rs and A_2A_Rs (Fredholm et al., 1999). Hippocampal A_2A_Rs are concentrated in synapses, where they selectively control synaptic plasticity processes (Rebola et al., 2008; Costenla et al., 2011; Temido-Ferreira et al., 2018) and play a prominent role in controlling the synaptic damage (Cunha et al., 2006; Silva et al., 2007, 2018; Canas et al., 2009b; Viana da Silva et al., 2016) that tightly correlates with memory impairment for instance in Alzheimer's disease (Selkoe, 2002; Coleman et al., 2004). Interestingly, we found that caffeine prevented the diabetes-induced loss of SNAP25 but not of synaptophysin. This is in agreement with previous observations suggesting that proteins of the SNARE complex are more robust indicators of synaptic dysfunction than proteins located in synaptic vesicles, such as synaptophysin (Reddy et al., 2005; Gao et al., 2006). We also observed that GK rats displayed a reduction of A_1_R immunoreactivity. This was not associated with a modification of A_1_R function controlling basal synaptic transmission in GK rats, in accordance with our observation that the modification of A_1_R density mostly occurs in total membranes. Since extra-synaptic A_1_Rs have recently been associated with modified information processing in cortical circuits (Florian et al., 2011; Serchov et al., 2015), future studies should focus on the possible role of A_1_Rs on memory performance through a control of neuron-glia communication.

In whole hippocampal membranes we observed a diabetes-induced reduction in A_1_R and increase of A_2A_R levels, which was normalized upon caffeine treatment. Therefore, limiting excessive activation of A_2A_Rs in extra-synaptic compartments, namely in glial cells, might also be a mechanism of neuroprotection by caffeine in T2D (Cunha, 2016). Indeed, apart from its synapto-protective action, caffeine had beneficial effects on T2D-induced astrogliosis, which emphasizes the potential neuroprotective role of glial A_2A_Rs (Daré et al., 2007), as reported in animal models of Alzheimer's (Matos et al., 2012) and Parkinson's disease (Yu et al., 2008), as well as exposure to LPS (Rebola et al., 2011) or glaucoma (Madeira et al., 2015).

It is important to stress that the present results do not exclude the possibility that the beneficial effects of chronic caffeine consumption might also involve the control of peripheral metabolism and circulating insulin concentration, such as via adenosine receptors in the pancreatic islet (e.g., Johansson et al., 2007; Töpfer et al., 2008; Salehi et al., 2009), or via regulation of peripheral metabolic rates and energy expenditure (van Dam and Hu, 2005; Greenberg et al., 2006; Higdon and Frei, 2006). It is of interest to note that GK rats chronically consuming caffeine displayed hyperglycemia but not hyperinsulinemia. The chronic caffeine treatment used in the present study was also previously found to improve peripheral insulin sensitivity and reduce circulating insulin concentration in aged rats (Guarino et al., 2013) and rats under diets rich in sugar or fat (Conde et al., 2012). In the brain, insulin and insulin-like growth factor 1 (IGF1) may be involved in regulating the presence of glucose carriers at the membrane of astrocytes (Fernandez et al., 2017), the expression of synaptic proteins and number of synapses (Chiu et al., 2008), the reactivity of astrocytes (e.g., Wilczak and De Keyser, 1997), and learning and memory processes (Zhao and Alkon, 2001). Notably, while insulin and insulin-sensitizing drugs have beneficial effect in dementia, it has also been proposed that persistent activation of insulin receptors could be the trigger for brain insulin resistance (e.g., Mullins et al., 2017). Therefore, further research is needed to understand the role of insulin in T2D-induced brain dysfunction.

In summary, long-term caffeine intake improved T2D-induced memory impairment, prevented astrogliosis, and ameliorated hippocampal synaptic degeneration in GK rats. Caffeine did not prevent T2D-associated metabolic modifications in the hippocampus. Nevertheless, it had an impact on metabolite concentrations in the hippocampus of both Wistar and GK rats.

Therefore, we conclude that the hippocampus is adaptable to different metabolic conditions, and that synaptic degeneration and astrogliosis rather than metabolic modifications contribute to diabetes-induced memory dysfunction. Finally, the present study also emphasizes the neuroprotective potential of chronic caffeine consumption as a prophylactic strategy to prevent memory impairment in T2D.

## Author Contributions

JD and RC designed the study. JD, CS, and HS performed experiments and analyzed data. JD wrote the manuscript. All authors contributed to the interpretation of the results and revised the manuscript.

### Conflict of Interest Statement

The authors declare that the research was conducted in the absence of any commercial or financial relationships that could be construed as a potential conflict of interest.

## Abbreviations

aCSF: artificial cerebrospinal fluid
Ala: alanine
Asc: ascorbate
Asp: aspartate
BBB: blood-brain-barrier
βHB: β-hydroxibutyrate
CADO: 2-chloroadenosine
CMR_glc_: cerebral metabolic rate of glucose consumption
Cr: creatine
CRLB: Cramér-Rao lower bound
DPCPX, 1: 3-dipropyl-8-cyclopentylxanthine
ELISA: enzyme-linked immunosorbent assay
fEPSP: field excitatory postsynaptic potential
GABA: γ-aminobutyrate
GFAP: glial fibrillary acidic protein
GK: Goto-Kakizaki
Glc: glucose
Gln: glutamine
Glu: glutamate
GPC: glycerophosphocholine
GSH: glutathione
I/O: input/output
Ins: *myo*-inositol
Lac: lactate
LTP: long-term potentiation
MAP2: microtubule-associated protein type 2
MRS: magnetic resonance spectroscopy
NAA: *N*-acetylaspartate
NAAG: *N*-acetylaspartatylglutamate
NMR: nuclear magnetic resonance
PCho: phosphocholine
PCr: phosphocreatine
PE: phosphoethanolamine
PSD95: postsynaptic density protein of 95 kDa
SCH58261: 2-(2-furanyl)-7-(2-phenylethyl)-7H-pyrazolo[4, 3-e][1, 2, 4]triazolo[1, 5-c]pyrimidin-5-amine
scyllo: *scyllo*-inositol
SNAP25: synaptosome-associated protein of 25 kDa
STZ: streptozotocin
Tau: taurine
VOI: volume of interest.
